# Revisiting the grand challenges in oral cancers: advances in biomarkers and treatment strategies

**DOI:** 10.3389/froh.2026.1875636

**Published:** 2026-06-08

**Authors:** Ricardo D. Coletta, Ahmed Al-Samadi, W. Andrew Yeudall, Tuula Salo

**Affiliations:** 1 Department of Oral Diagnosis and Graduate Program in Oral Biology, School of Dentistry, University of Campinas, Piracicaba, Brazil; 2 Department of Oral and Maxillofacial Diseases, Clinicum, Faculty of Medicine, University of Helsinki, Helsinki, Finland; 3 Institute of Dentistry, School of Medicine, Faculty of Health Sciences, University of Eastern Finland, Kuopio, Finland; 4 Department of Oral and Maxillofacial Diseases, and iCAN Digital Precision Cancer Medicine Flagship, University of Helsinki, Helsinki, Finland; 5 Department of Oral Biology, The Dental College of Georgia at Augusta University, and Georgia Cancer Center, Augusta, GA, United States; 6 Research Unit of Population Health, Department of Oral Pathology, University of Oulu and Oulu University Hospital, Oulu, Finland; 7 Medical Research Center, Oulu University Hospital, Oulu, Finland; 8 Department of Oral and Maxillofacial Diseases, Translational Immunology Program (TRIMM), and iCAN Digital Precision Cancer Medicine Flagship, University of Helsinki, Helsinki, Finland; 9 Department of Pathology, Helsinki University Hospital, Helsinki, Finland

**Keywords:** biomarkers, oral squamous cell carcinoma, precision oncology, prognosis, therapeutic strategies

## Abstract

Although a wide range of molecular and histopathological biomarkers has been explored for oral squamous cell carcinoma (OSCC), few have reached the level of validation needed for routine clinical application. Updates to the TNM staging system and accumulating evidence for emerging histopathological parameters may enhance prognostic accuracy, but robust prospective studies are still required. Progress in genomics, transcriptomics, proteomics, and liquid biopsy technologies has identified several promising candidates, however methodological variations and the limited number of validation studies continue to slow their clinical adoption. Current OSCC treatment remains centered on surgery, radiotherapy, and chemotherapy, with targeted therapies and immunotherapy drugs offering only modest benefits. Emerging precision oncology strategies and patient-derived drug testing platforms hold potential, though technical and biological barriers still limit their widespread application. In this expert perspective, the discussion highlights advances in emerging biomarkers, evaluates their readiness for clinical application, and examines evolving treatment strategies. It also outlines key translational challenges and future directions that may support the development of more personalized OSCC management.

## Introduction

Oral squamous cell carcinoma (OSCC), arising from the epithelial mucosa of the oral cavity, accounts for more than 90% of intraoral malignant neoplasms. It represents a major global health problem with substantial social and economic burdens, driven by its high aggressiveness, elevated recurrence rates, and persistently poor survival outcomes, which have shown little improvement over recent decades. Trends indicate a rising incidence of OSCC, particularly in countries with lower human development index levels, as well as among women and younger individuals, driven largely by shifting lifestyle factors. These unfavorable outcomes stem from the fact that most cases are diagnosed at an advanced stage and from the lack of reliable biomarkers capable of identifying highly aggressive tumors, predicting patient prognosis, and guiding individualized treatment decisions. In addition, the clinical impact of novel cancer therapies remains limited in oral cancer, as many of the advances seen in other tumor types have not yet translated effectively to patients with OSCC.

In 2020, with the launch of Frontiers in Oral Health, we had the opportunity to publish the article “Grand Challenges in Oral Cancers*”* (1), in which we outlined what we viewed as the major research challenges facing the field amid rapid scientific and technological change. In 2024, we revisited the topic, reflecting on developments in the prevalence of oral cancer, its risk factors and emerging prevention strategies (2). In this article, we extend those reflections by examining current prospects in biomarkers, summarizing recent advances and highlighting the key barriers that still hinder their clinical translation, and in treatment, discussing established protocols as well as emerging targeted-therapy and immunotherapy drugs.

### Biomarkers: advances and barriers to clinical implementation

With rapid evolution, biomarkers have become central to guiding clinical decision-making in oncology. They ideally have a predictive function, helping determine whether a tumor is likely to benefit from a specific treatment, or a prognostic role, offering insight into the expected disease trajectory and patient outcome. Although numerous biomarkers have been proposed for OSCC, none has yet reached sufficient validation for routine clinical use. As a result, TNM clinical staging remains the most dependable tool for prognostication and treatment planning. The most recent edition of the TNM system introduced two major refinements: depth of invasion was added to the T category, and extranodal extension was incorporated into the N category. These updates appear to improve risk stratification and move the field toward more individualized care, as shown by a systematic review including 10 studies (3), and a second review published in 2024 examining 13 studies (4). However, most supporting evidence comes from retrospective restaging of cases originally classified under older TNM versions, which may introduce bias. Although the published findings are encouraging, we still need prospective studies with long-term follow-up to determine the true clinical value of the revised staging system.

Several histopathological features, such as perineural invasion, lymphovascular invasion and margin status, are well-recognized as markers of more aggressive behavior (5), but their incorporation into decision-making varies considerably among clinicians. In the last few years, a number of other histopathological characteristics, most of them identifiable on standard hematoxylin and eosin-stained sections, have gained great attention due to the strong and consistent results derived from many independent studies and systematic reviews, but they are still not part of routine evaluation in OSCC. Among these emerging histopathological characteristics are tumor budding, worst pattern of invasion, tumor-stroma ratio, and the density of tumor-infiltrating lymphocytes (6). Interestingly, the most recent edition of the WHO Classification of Head and Neck Tumours (2024) underscores the need for updates in this area, and highlights the relevance of these features in refining prognostic assessment for OSCC. It is anticipated that the expert panel contributing to the WHO Classification of Head and Neck Tumours may acknowledge the substantial evidence supporting the prognostic value of these emerging histopathological parameters in OSCC, which overcomes the shortcomings of the traditional grading systems more effectively, and may recommend that pathologists include these features in histopathological reports for both biopsies and surgical specimens. Applying these histopathological parameters to biopsies will be a significant challenge and will depend heavily on clinicians' performance.

With the advent of high-throughput technologies capable of surveying wide molecular alterations, a broad range of DNA-, RNA-, protein- and metabolism-based biomarkers has been explored for prognostic purposes. These include gene mutations, single-nucleotide variations, copy-number changes, RNA expression profiles (both mRNA and non-coding RNA), as well as proteomic and metabolomic landscapes. These technologies have enabled the identification of prognostic signatures, which are expected to be more effective than single-marker approaches. Several systematic reviews have highlighted the association of microRNA expression with OSCC prognosis. For example, a meta-analysis of 622 patients indicated that overexpression of miR-21 is associated with a worse prognosis (7), while another one indicated that elevated levels of miR-31 was also related to decreased overall survival (8). Liquid biopsy strategies, using either serum or saliva, are also potentially powerful tools for OSCC diagnostics and prognostics. A systematic review revealed that the presence of circulating tumor (ct) DNA in patients’ blood was predictive of both poorer overall survival and progression-free survival, whereas ctHPV-DNA (human papillomavirus DNA) was associated with decreased overall survival (9). A recent exploratory systematic review of potential salivary biomarkers identified lactate dehydrogenase amongst several other proteins whose increased expression was associated with disease outcome and/or malignant transformation (10).

Protein-level markers assessed by immunohistochemistry have historically dominated the field and continue to represent a major focus of biomarker research. Many potential markers have been reported, but most have not been incorporated into routine OSCC practice due to limited validation. Others, such as p53 and Ki-67, have been extensively studied yet remain excluded because of inconsistent findings. At present, programmed cell death protein 1 (PD-1) and programmed cell death-ligand 1 (PD-L1, also known as CD274 and B7-H1) are receiving the greatest scientific attention because of their relevance to immune-checkpoint inhibitors used in immunotherapy. PD-1 is an immune checkpoint receptor expressed by activated lymphocytes, which binds to PD-L1 on the surface of both normal (mainly inflammatory cells) and tumor cells. Tumor cells overexpressing PD-L1 tend to escape immune attack, and monoclonal antibodies blocking PD-1 restore antitumor immunity, demonstrating efficacy across a wide range of cancers (more details below). Although expression of PD-L1 and PD-1 are clinically relevant for immunotherapy, current evidence remains ambiguous, with several studies and systematic reviews failing to support their use as reliable prognostic markers for survival (11, 12). These inconsistencies are likely related to the lack of standardization in sample selection, assessment methods, antibodies employed, scoring metrics, and cutoff parameters. Due to their importance for immunotherapy response, studies with large cohorts from multicenter trials, integrating multiple clinical and histopathological markers and taking into account these critical gaps will definitely elucidate the role of the PD-1/PD-L1 axis in OSCC prognosis more clearly.

Although numerous potential biomarkers for OSCC have been reported, most published studies fall short of high methodological standards in their planning, execution, and reporting, resulting in limited high-quality evidence. In addition, key findings have rarely been validated consistently in large, prospective, multi-center cohorts, which would strengthen the evidence base and facilitate clinical translation. Several guidelines have been developed to improve biomarker research, with the REMARK (Reporting Recommendations for Tumor Marker Prognostic Studies) criteria still regarded as the gold standard. Moreover, though these observations are promising, a grand challenge will be to develop a robust panel of validated biomarkers, integrating AI-based analytical approaches, that will accurately predict disease status, therapeutic response and clinical outcome.

### Therapeutic approaches: current practices and evolving strategies

Oral cancer treatment remains an area where major therapeutic breakthroughs have been limited in recent years, despite extensive research efforts. Current management continues to rely largely on conventional approaches, with surgery as the cornerstone of treatment, followed by radiotherapy with or without chemotherapy (13). Although advances in molecular oncology have transformed the treatment landscape of several malignancies through the introduction of targeted therapies and immunotherapies, their impact on oral cancer has been comparatively modest. To date, only a small number of such agents have been approved for oral cancer, such as the epidermal growth factor receptor (EGFR) inhibitor cetuximab and the PD-1 inhibitors nivolumab and pembrolizumab. This limited range of therapies contrasts sharply with other malignancies such as lung, breast, and colorectal cancers, where numerous targeted and immunotherapeutic agents are available.

Radiotherapy represents the second major treatment modality after surgery and plays a critical role in both curative and adjuvant settings. It can be administered as a primary treatment, preoperatively or postoperatively, either alone or in combination with chemotherapy, targeted therapy, or immunotherapy. Although treatment protocols vary across countries and institutions, commonly accepted indications for postoperative radiotherapy include positive surgical margins, multiple involved lymph nodes, presence of extranodal extension, bone invasion, lymphovascular inasion, and perineural invasion (14). Radiotherapy is delivered using techniques such as conventional external beam radiotherapy, intensity-modulated radiation therapy, and brachytherapy, each with distinct advantages and limitations in terms of precision, toxicity, long-term side effects, and clinical outcomes.

Chemotherapy remains a key component of oral cancer treatment, typically administered in combination with radiotherapy, particularly in patients with locally advanced, recurrent or metastatic disease. Commonly used agents include cisplatin, 5-fluorouracil, docetaxel, and carboplatin, with the combination of cisplatin, 5-fluorouracil, and docetaxel (TPF) currently regarded as the most recommended regimen. Another frequently used regimen is PF (cisplatin + 5-fluorouracil), which offers lower toxicity but a modest survival disadvantage compared to TPF (15). Although chemotherapy generally produces more consistent responses than currently available targeted or immunotherapies, its clinical utility is limited by significant toxicity due to lack of specificity for cancer cells, resulting in adverse effects that compromise treatment adherence and quality of life. The acquisition of chemoresistance, particularly with cisplatin and 5-fluorouracil when used as single agents, represents another major drawback (16).

Cetuximab has been used in patients with locally advanced disease in combination with radiotherapy, as well as in recurrent and/or metastatic settings in combination with platinum-based chemotherapy, followed by maintenance therapy until disease progression. These regimens have demonstrated improvements in locoregional control and survival outcomes, particularly when combined with radiotherapy, although the magnitude of benefit remains modest and patient selection is critical (17). In a prospective trial comparing cetuximab plus radiotherapy with radiotherapy alone for head and neck squamous cell carcinoma (HNSCC) in the postoperative setting, Machtay et al (18). reported no difference in overall survival but observed a statistically significant improvement in disease-free survival, without an increase in long-term toxicity. In a recent clinical study conducted across six oncology centers in Croatia, the EXTREME regimen, combining cetuximab with cisplatin or carboplatin and 5-fluorouracil, achieved an objective response rate of 21% and a disease control rate of 63% in patients with recurrent and/or metastatic HNSCC, with side effects generally well tolerated (19). One of the main advantages of cetuximab is its generally more favorable toxicity profile compared with conventional chemotherapeutic agents, largely due to its targeted mechanism of action. However, resistance to cetuximab frequently develops during treatment, which limits its long-term effectiveness.

Immune checkpoint inhibitors, particularly nivolumab and pembrolizumab, are currently used in recurrent and metastatic disease, either as monotherapy or in combination with chemotherapy (20). Following the KEYNOTE-048 randomized, open-label phase 3 trial, the combination of pembrolizumab with platinum and 5-fluorouracil is now regarded as an appropriate first-line option for recurrent or metastatic HNSCC. In patients with PD-L1-positive disease, pembrolizumab monotherapy is also considered a suitable first-line treatment approach (21). Notably, in 2025, the U.S. Food and Drug Administration approved pembrolizumab for patients with unresectable, locally advanced HNSCC (U.S. Food and Drug Administration statement on June 12, 2025). This represented the first major regulatory approval for an immunotherapy in this disease setting in several years. Despite the immunogenic nature of HNSCC, including oral cancer, response rates to PD-1 blockade remain relatively low, with no proven efficacy in the curative setting to date, as revealed by a systematic review of phase-3 clinical trials (22). Moreover, although survival outcomes are improved, the marginal improvement should be weighed against the cost of these therapeutic agents and the quality of life of patients.

Due to the limited number of effective approved drugs for oral cancer, implementing a truly personalized treatment approach remains challenging. While precision oncology strategies in cancers such as breast and colorectal cancer are guided by actionable mutations in genes such as *PIK3CA*, *KRAS* and *BRAF* (23), similar approaches in oral cancer are hindered by the scarcity of validated targets and approved drugs. A recent study examined the clinical outcomes of patients treated with paclitaxel with or without the pan-PI3K inhibitor buparlisib (24). Notably, patients whose cancers were positive for tumor-infiltrating lymphocytes and showed activation of either PI3K or NOTCH signaling pathways had improved overall survival when buparlisib was included in the treatment regimen. Another promising but still largely experimental approach is personalized drug testing using patient-derived tumor samples. This field has expanded considerably in recent years, with numerous *in vitro* and *in vivo* models, such as organoids and patient-derived xenografts, proposed across different cancer types, including oral cancer [for more details see (25)]. While results have generally been encouraging, clinical translation in solid tumors remains limited, in contrast to more successful applications in hematological malignancies (26). In oral cancer, several factors contribute to this challenge, including difficulties in processing tumor tissues, a high risk of bacterial and fungal contamination, overgrowth of fibroblasts, and the relatively small number of samples tested in most studies.

## Conclusion

The discovery of effective biomarkers for predicting the risk of recurrence and survival and choosing the optimal therapy is largely an unmet need for OSCC, reflecting in a dismal prognosis of the patients. Despite significant progress in recent decades with the development of targeted therapies, immunotherapies, and the use of molecular biomarkers to guide patient treatment across several tumor types, this knowledge has yet to be effectively translated into routine clinical practice for OSCC. Addressing this challenge will require multidisciplinary efforts focused on the development and testing of novel anticancer agents, identification of predictive biomarkers to optimize the use of existing therapies, and further advancement of personalized drug testing platforms. Achieving meaningful progress will also depend on sustained funding and close collaboration between clinicians, researchers, and the pharmaceutical industry, with the ultimate goal of improving survival and quality of life for patients with oral cancer. Looking ahead, continued innovation in precision oncology, functional drug testing, and AI-enabled biomarker discovery offers a promising path toward more individualized and effective treatment strategies for OSCC.

## Data Availability

The datasets presented in this article are not readily available because this is a narrative review, based on previously published studies and does not include any new data. Requests to access the datasets and further inquiries can be directed to the corresponding author.
